# Supplementary material for: Phenotypic but not genetically predicted heart rate variability associated with all-cause mortality
Source: Commun Biol. 2023 Oct 6;6:1013. doi: 10.1038/s42003-023-05376-y (PMC10558565; doi:10.1038/s42003-023-05376-y)
Supplement: Supplementary file 2 — Supplementary Information [file 42003_2023_5376_MOESM2_ESM.pdf]

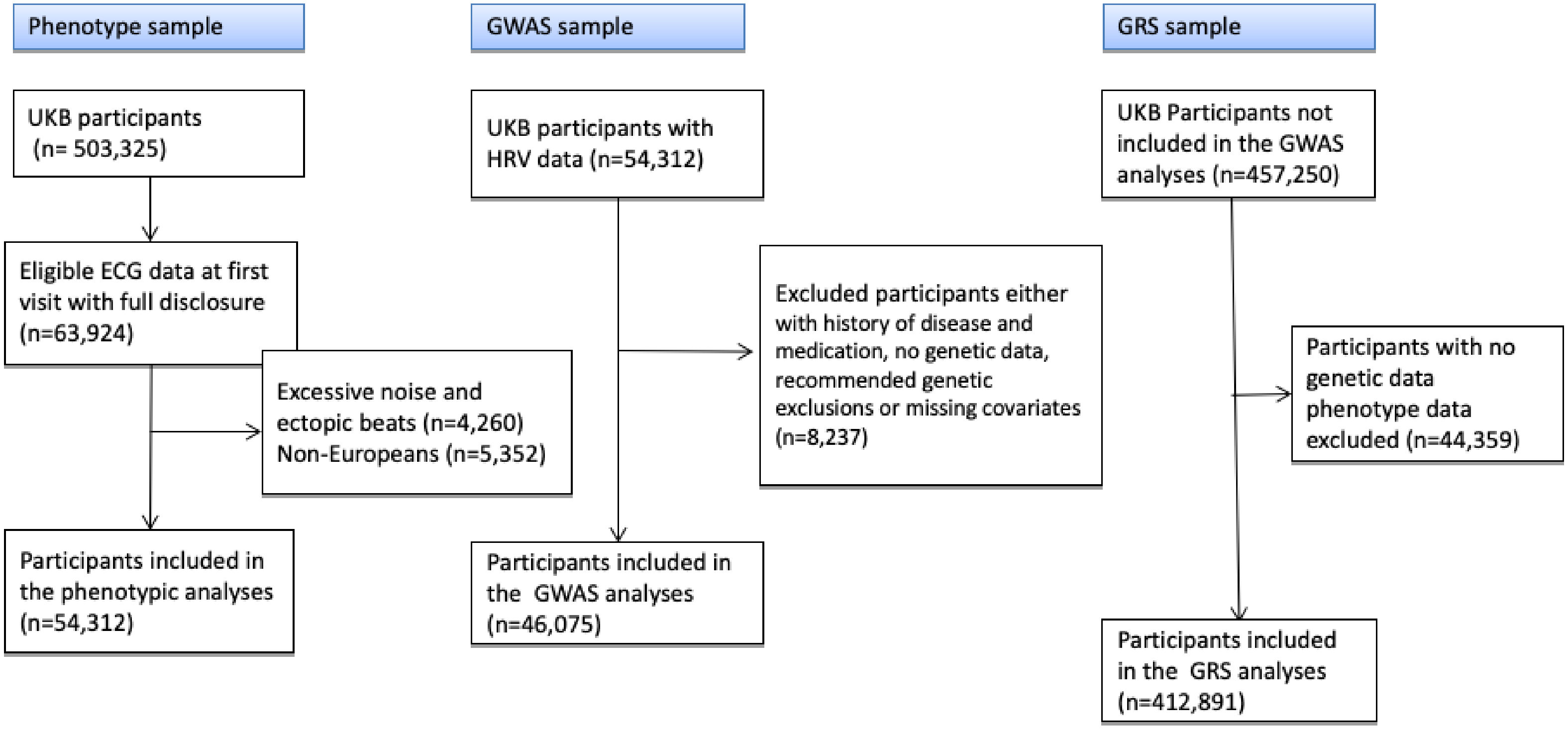

Supplementary Figure 1: Flowchart of the sample selection for the phenotypic, GWAS, and GRS analyses. The number of exclusions for the GWAS sample were sequential and there might be some overlap. Participants in the phenotype sample were also part of the GWAS sample however some participants in the GWAS sample were excluded due to further exclusion criteria like absence of genetic data and diagnoses of diseases and use of medications that influence heart rate variability. Participants excluded based on diseases and medications if they had a diagnosis of angina, myocardial infarction, heart failure or used anti-depressant medication, digoxin, atropine or acetylcholinesterase. UKB: UK Biobank; HRV: Heart rate variability; ECG: Electrocardiogram; GWAS: Genome-wide association study; GRS: genetic risk score.

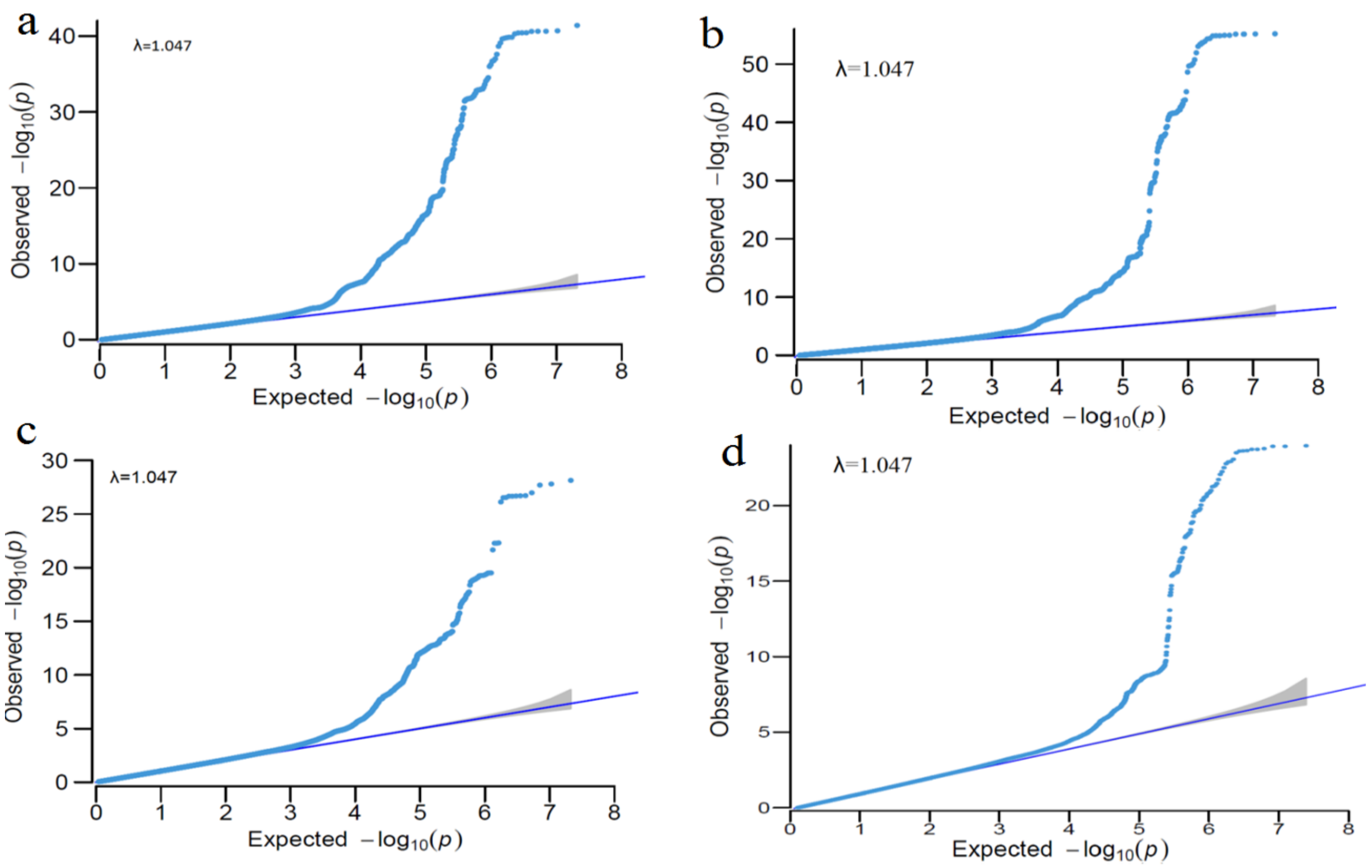

Supplementary Figure 2: **Quantile-quantile-plots of the HRV GWAS results of (a) RMSSD, (b) RMSSDc, (c) SDNN, and (d) SDNNc**. Expected –log10(p-values) assuming a normal distribution are shown on the x-axis for each SNPs (blue dots). The y-axis depicts the observed –log10(pvalues). The blue line indicates the null hypothesis of no true association, and the grey shaded area indicates the 95% confidence interval. RMSSD, root mean square of successive differences; RMSSDc, heart rate corrected root mean square of successive differences; SDNN, SD of normal-to-normal intervals; and SDNNc, heart rate corrected SD of normal-to-normal intervals

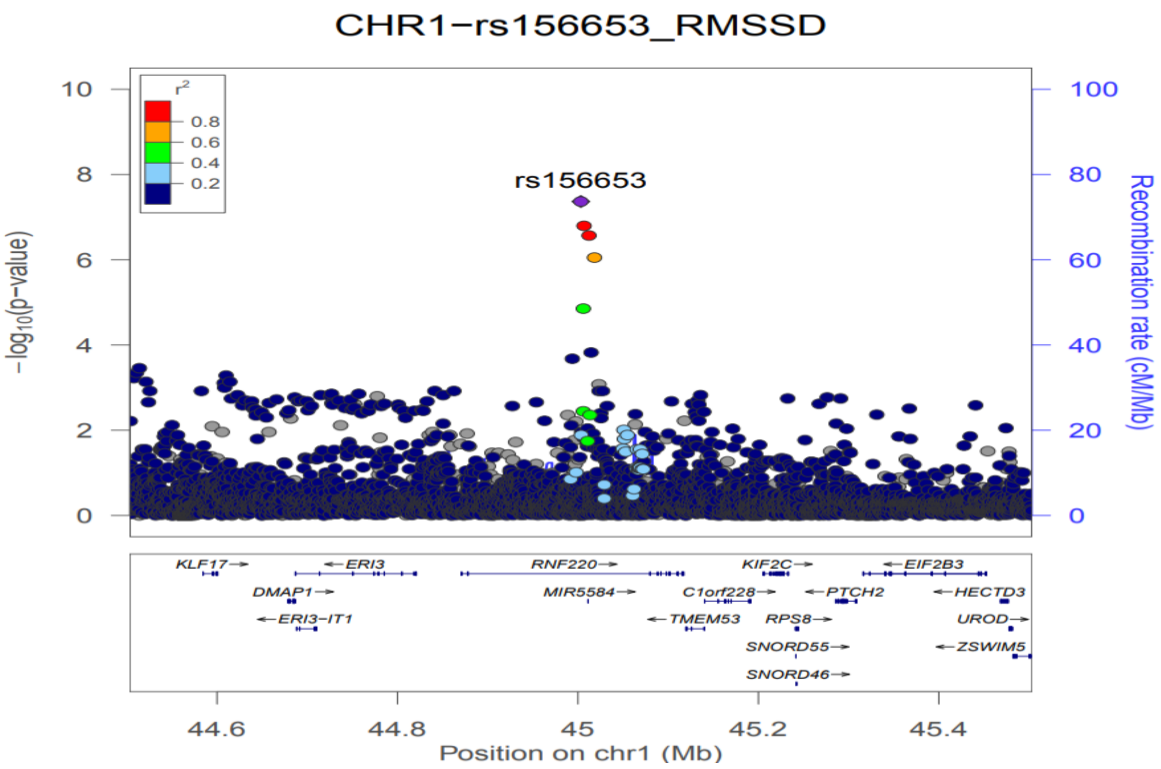

Supplementary Figure 3. Regional association plots for SNP rs156653. Chromosome number and HRV trait are indicated in the top line. The x-axis depicts a 1Mb region, 500kb either side of the sentinel variant (purple diamond) and the bottom panel show genes located within the region. The left y-axis shows −log10 p-values for individual SNPs and the light blue line on the right Y-axis indicates the recombination rates. Pairwise LD (r^2) with the sentinel variant is based on 1000 Genomes European reference samples and is described using the color coding.

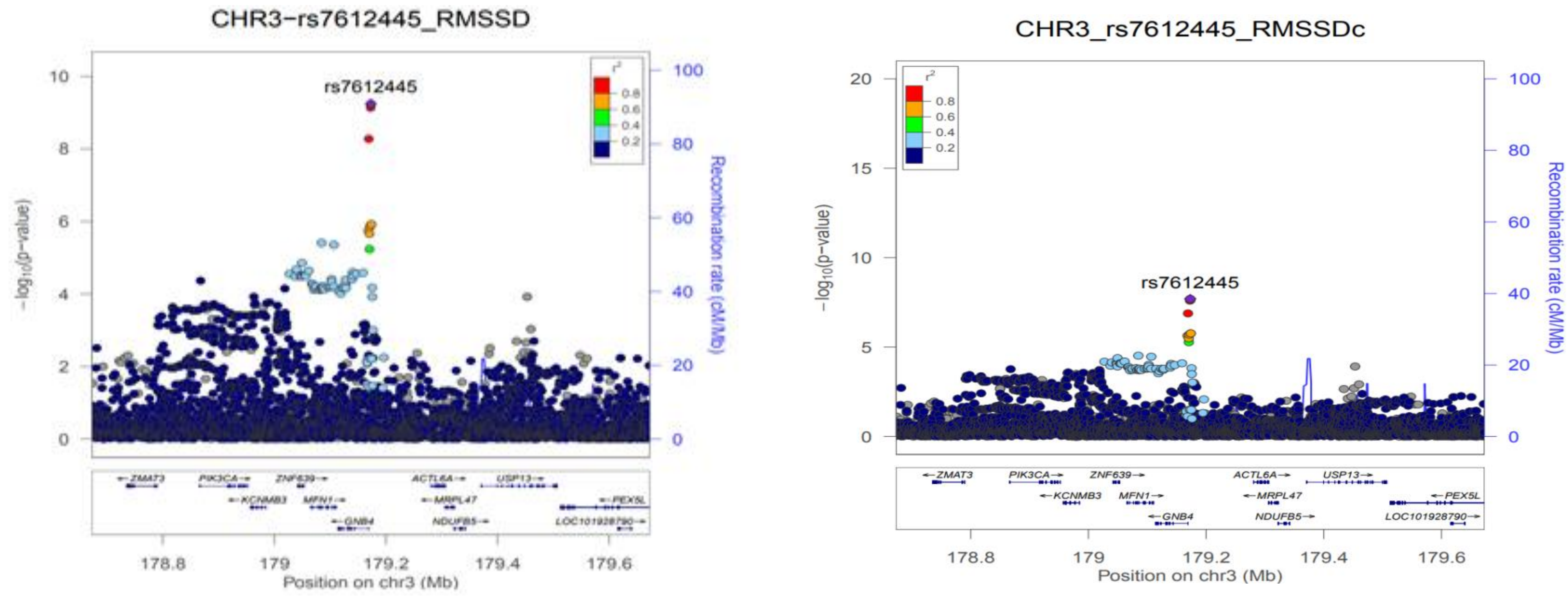

Supplementary Figure 4. Regional association plots for SNP rs7612445. Chromosome number and HRV trait are indicated in the top line. The x-axis depicts a 1Mb region, 500kb either side of the sentinel variant (purple diamond) and the bottom panel show genes located within the region. The left y-axis shows −log10 p-values for individual SNPs and the light blue line on the right Y-axis indicates the recombination rates. Pairwise LD (r^2) with the sentinel variant is based on 1000 Genomes European reference samples and is described using the color coding.

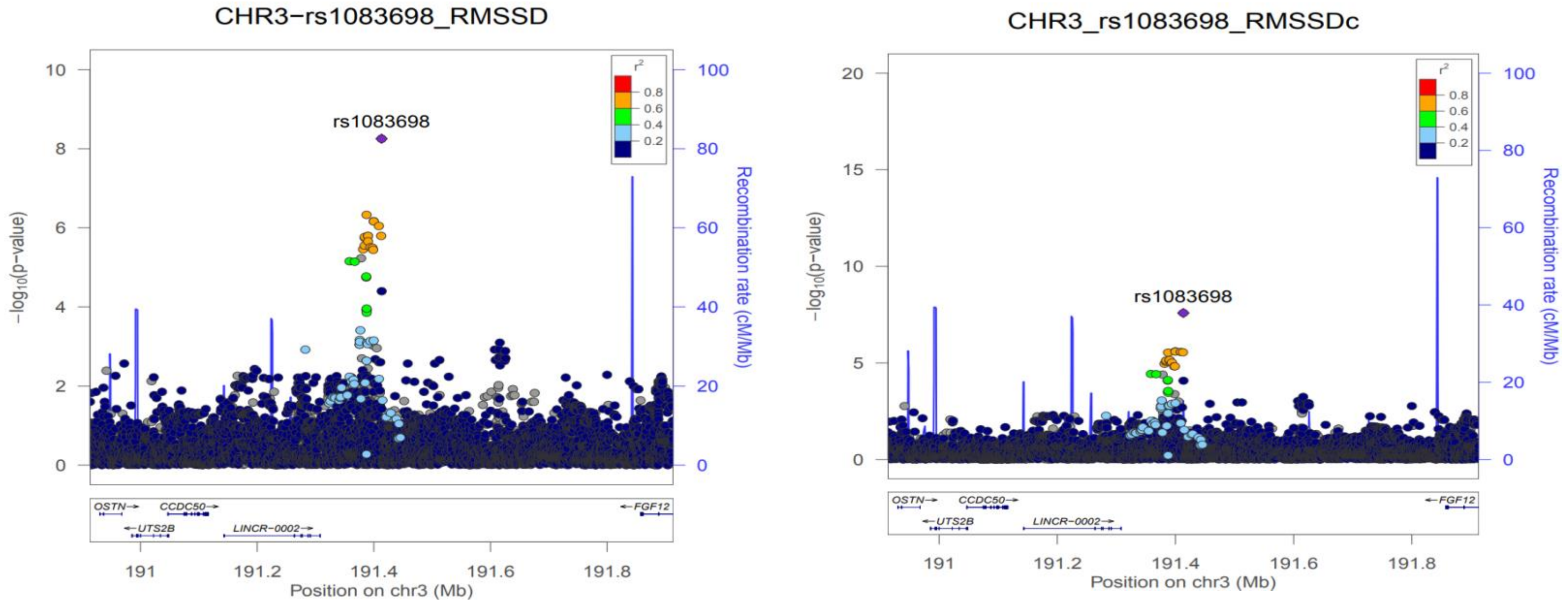

Supplementary Figure 5. Regional association plots for SNP rs1083698. Chromosome number and HRV trait are indicated in the top line. The x-axis depicts a 1Mb region, 500kb either side of the sentinel variant (purple diamond) and the bottom panel show genes located within the region. The left y-axis shows −log10 p-values for individual SNPs and the light blue line on the right Y-axis indicates the recombination rates. Pairwise LD (r^2) with the sentinel variant is based on 1000 Genomes European reference samples and is described using the color coding.

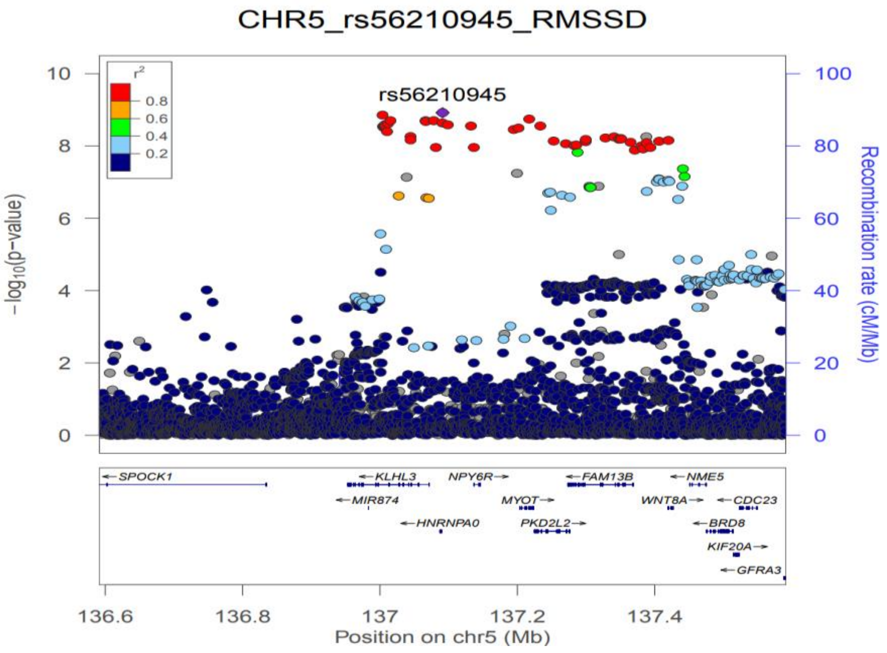

Supplementary Figure 6. Regional association plots for SNP rs56210945. Chromosome number and HRV trait are indicated in the top line. The x-axis depicts a 1Mb region, 500kb either side of the sentinel variant (purple diamond) and the bottom panel show genes located within the region. The left y-axis shows −log10 p-values for individual SNPs and the light blue line on the right Y-axis indicates the recombination rates. Pairwise LD (r^2) with the sentinel variant is based on 1000 Genomes European reference samples and is described using the color coding.

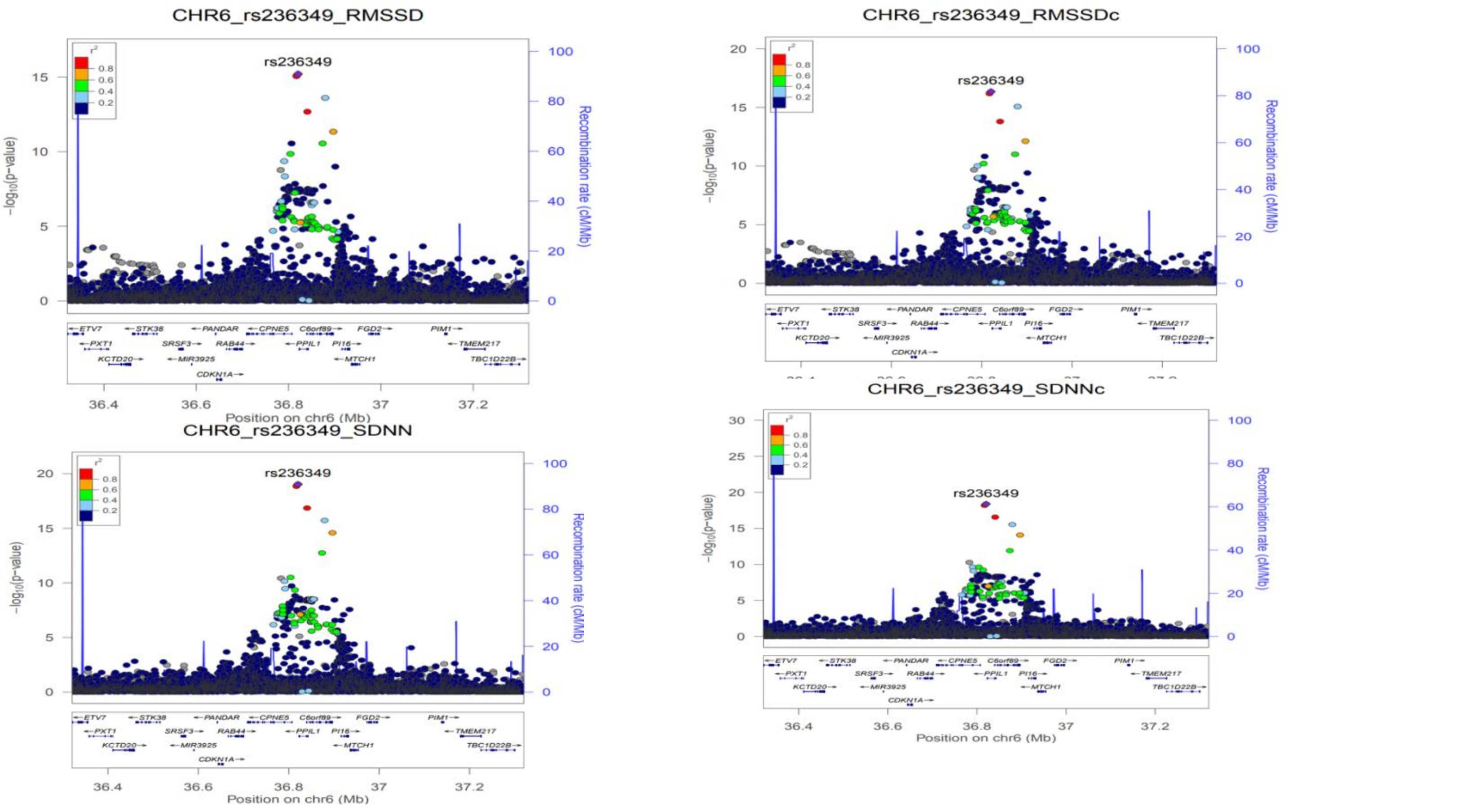

Supplementary Figure 7. Regional association plots for SNP rs236349. Chromosome number and HRV trait are indicated in the top line. The x-axis depicts a 1Mb region, 500kb either side of the sentinel variant (purple diamond) and the bottom panel show genes located within the region. The left y-axis shows −log10 p-values for individual SNPs and the light blue line on the right Y-axis indicates the recombination rates. Pairwise LD (r^2) with the sentinel variant is based on 1000 Genomes European reference samples and is described using the color coding.

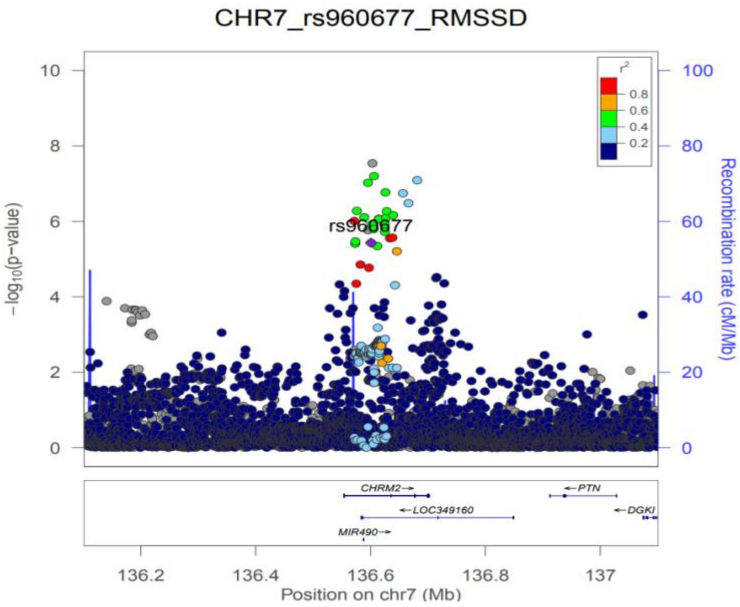

Supplementary Figure 8. Regional association plots for SNP rs71784944. Chromosome number and HRV trait are indicated in the top line. The x-axis depicts a 1Mb region, 500kb either side of the sentinel variant (purple diamond) and the bottom panel show genes located within the region. The left y-axis shows −log10 p-values for individual SNPs and the light blue line on the right Y-axis indicates the recombination rates. Pairwise LD (r^2) with the sentinel variant is based on 1000 Genomes European reference samples and is described using the color coding. Since rs71784944 is not available in the 1000 Genomes, we used a proxy SNP, rs960677, for the regional association plot.

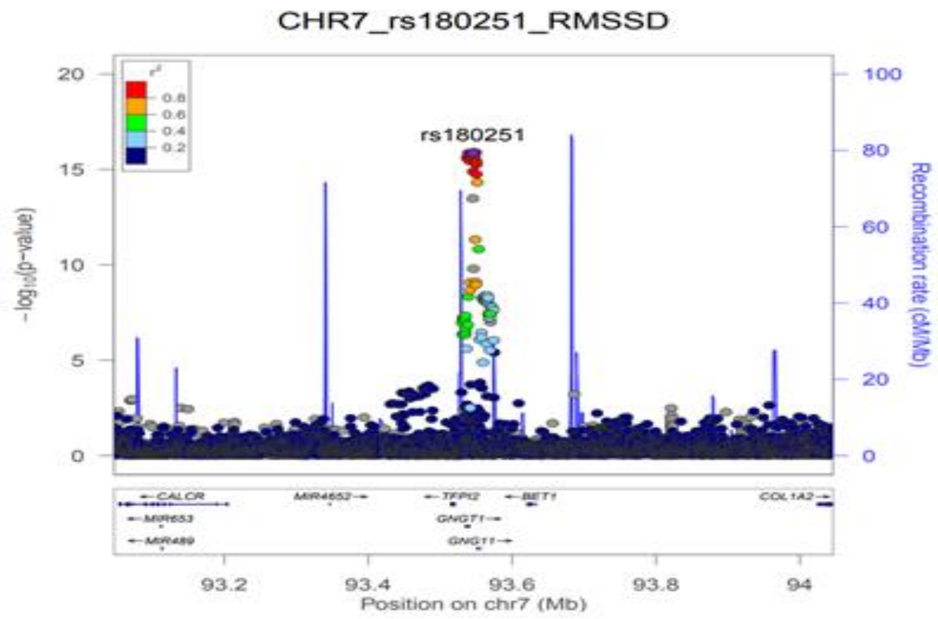

Supplementary Figure 9. Regional association plots for SNP rs180251. Chromosome number and HRV trait are indicated in the top line. The x-axis depicts a 1Mb region, 500kb either side of the sentinel variant (purple diamond) and the bottom panel show genes located within the region. The left y-axis shows −log10 p-values for individual SNPs and the light blue line on the right Y-axis indicates the recombination rates. Pairwise LD (r^2) with the sentinel variant is based on 1000 Genomes European reference samples and is described using the color coding.

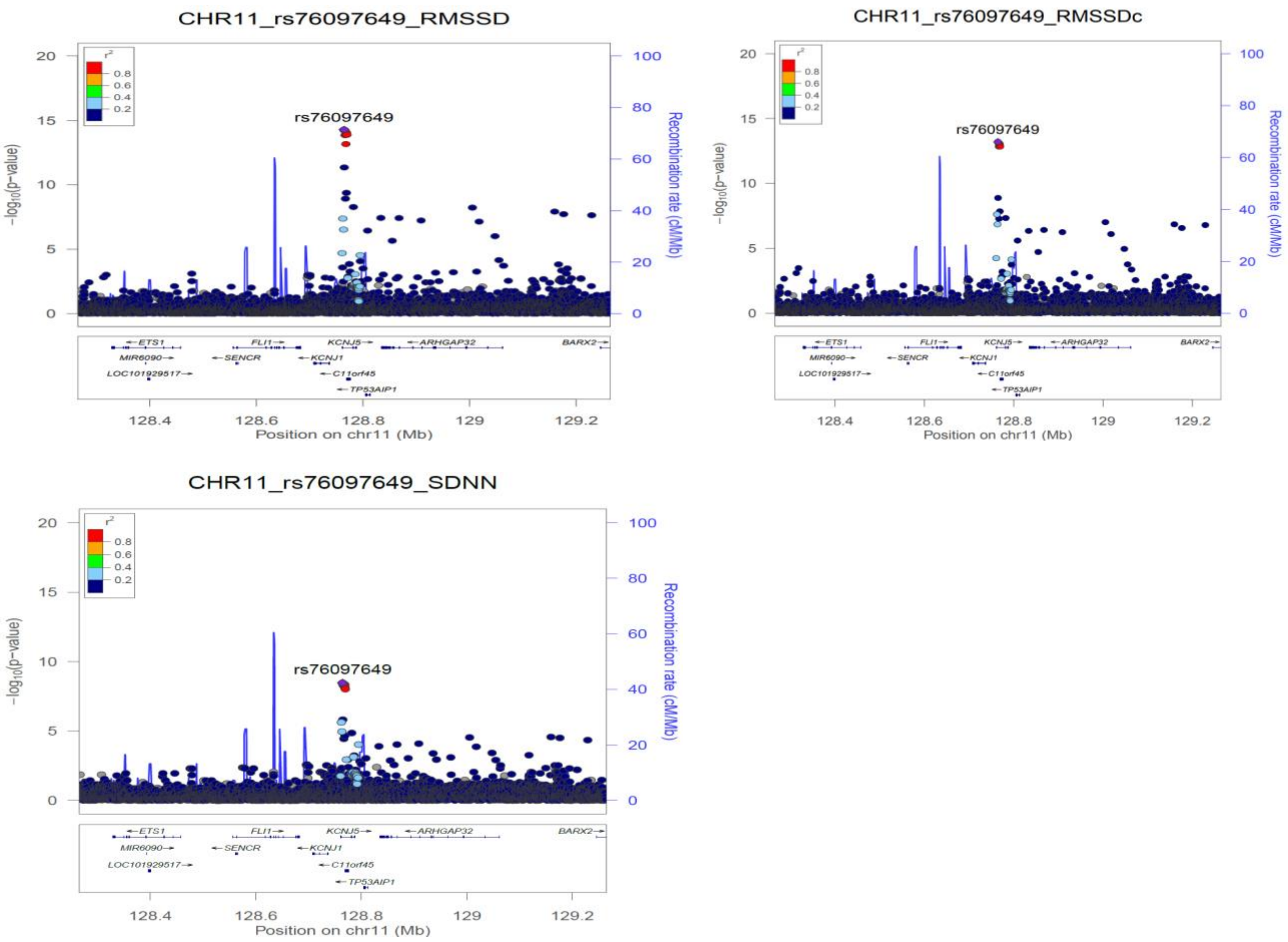

Supplementary Figure 10. Regional association plots for SNP rs76097649. Chromosome number and HRV trait are indicated in the top line. The x-axis depicts a 1Mb region, 500kb either side of the sentinel variant (purple diamond) and the bottom panel show genes located within the region. The left y-axis shows −log10 p-values for individual SNPs and the light blue line on the right Y-axis indicates the recombination rates. Pairwise LD (r^2) with the sentinel variant is based on 1000 Genomes European reference samples and is described using the color coding.

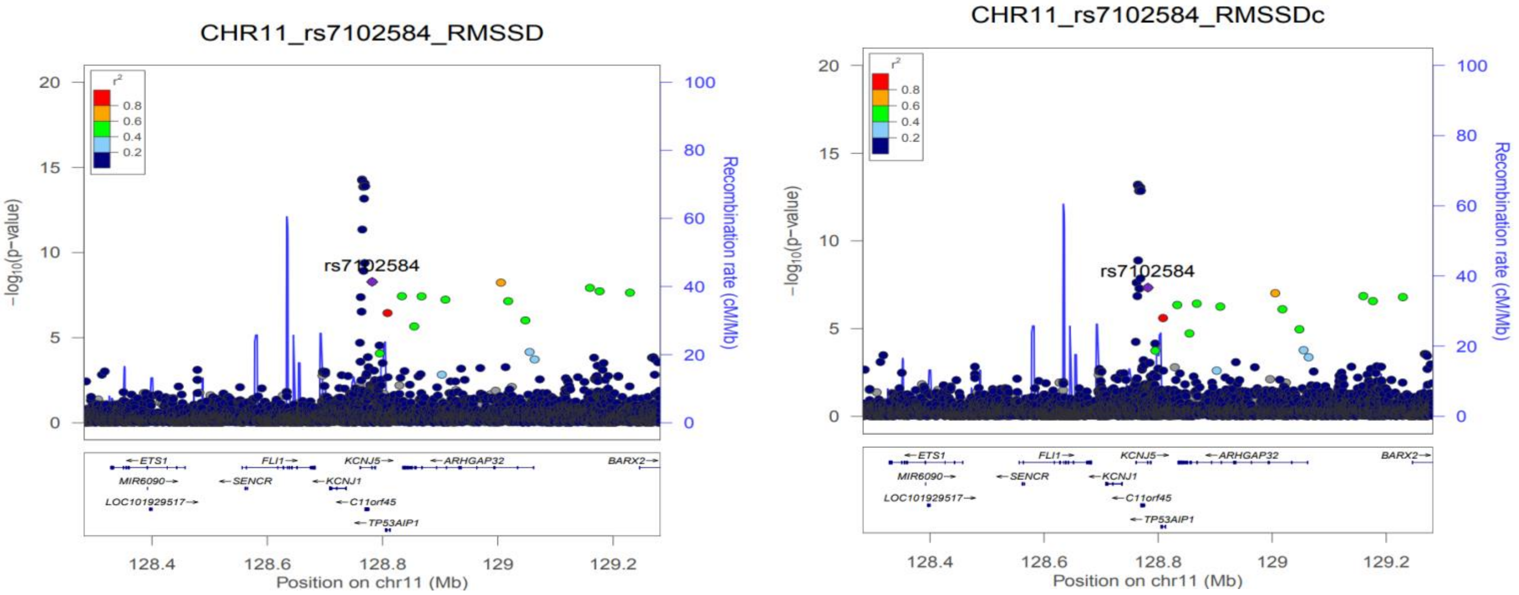

Supplementary Figure 11. Regional association plots for SNP rs7102584. Chromosome number and HRV trait are indicated in the top line. The x-axis depicts a 1Mb region, 500kb either side of the sentinel variant (purple diamond) and the bottom panel show genes located within the region. The left y-axis shows −log10 p-values for individual SNPs and the light blue line on the right Y-axis indicates the recombination rates. Pairwise LD (r^2) with the sentinel variant is based on 1000 Genomes European reference samples and is described using the color coding.

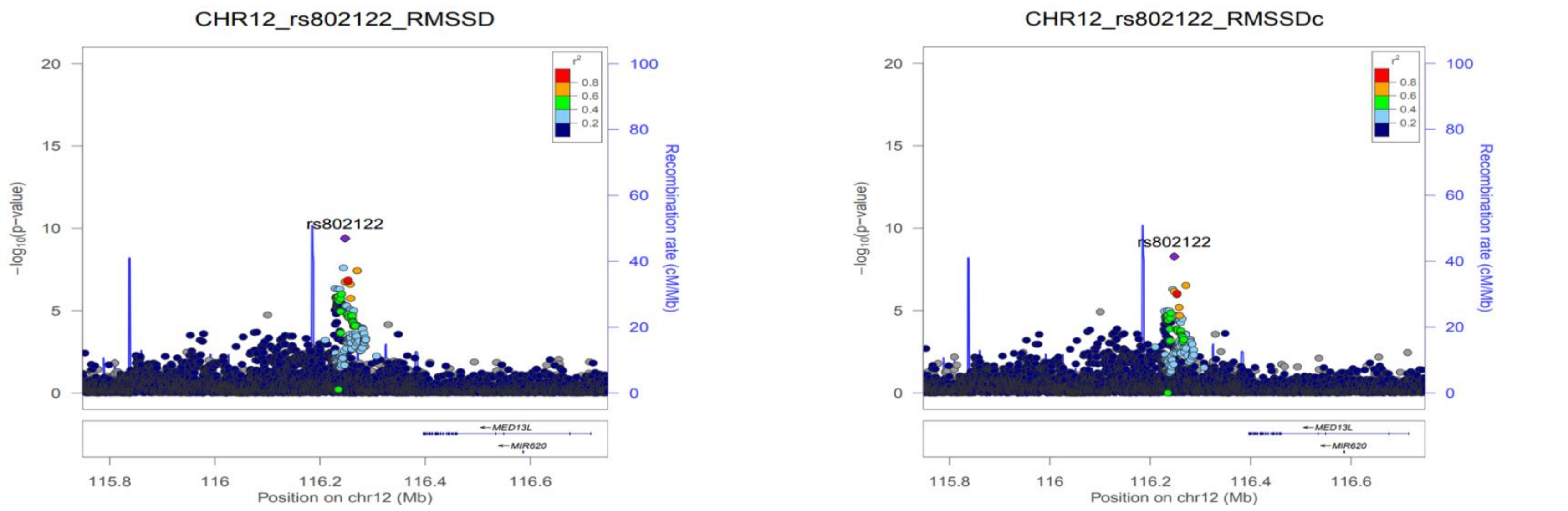

Supplementary Figure 12. Regional association plots for SNP rs802122. Chromosome number and HRV trait are indicated in the top line. The x-axis depicts a 1Mb region, 500kb either side of the sentinel variant (purple diamond) and the bottom panel show genes located within the region. The left y-axis shows −log10 p-values for individual SNPs and the light blue line on the right Y-axis indicates the recombination rates. Pairwise LD (r^2) with the sentinel variant is based on 1000 Genomes European reference samples and is described using the color coding.

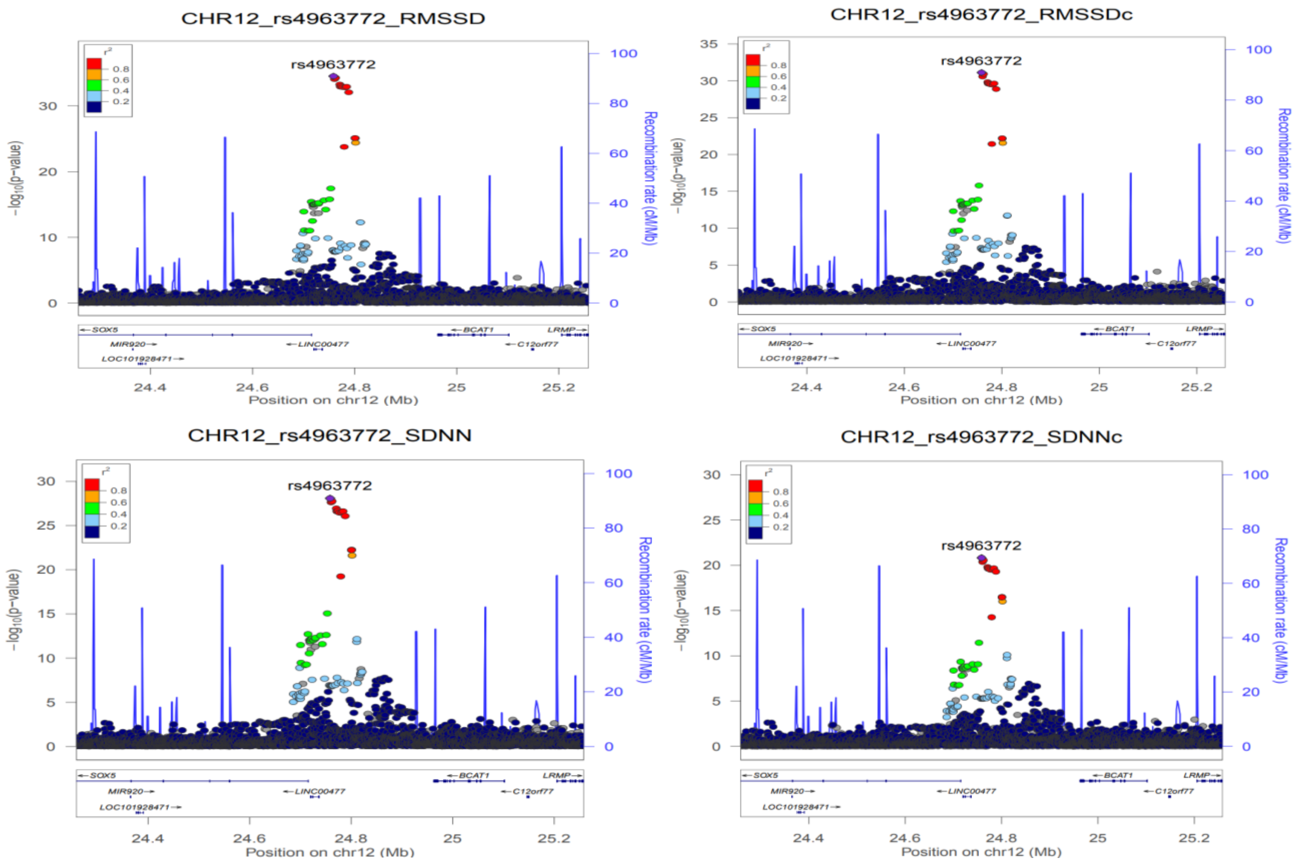

Supplementary Figure 13. Regional association plots for SNP rs4963772. Chromosome number and HRV trait are indicated in the top line. The x-axis depicts a 1Mb region, 500kb either side of the sentinel variant (purple diamond) and the bottom panel show genes located within the region. The left y-axis shows −log10 p-values for individual SNPs and the light blue line on the right Y-axis indicates the recombination rates. Pairwise LD (r^2) with the sentinel variant is based on 1000 Genomes European reference samples and is described using the color coding.

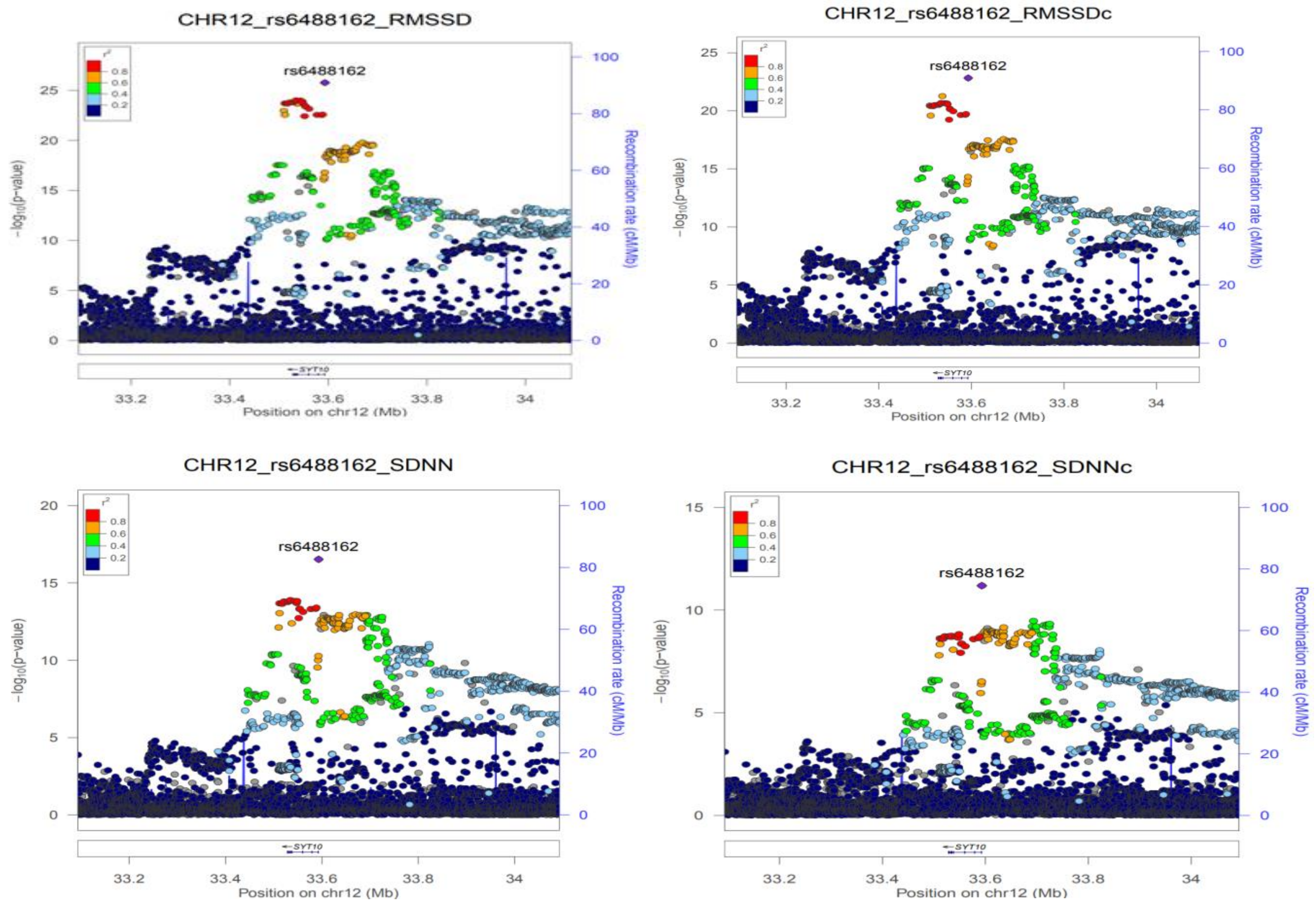

Supplementary Figure 14. Regional association plots for SNP rs6488162. Chromosome number and HRV trait are indicated in the top line. The x-axis depicts a 1Mb region, 500kb either side of the sentinel variant (purple diamond) and the bottom panel show genes located within the region. The left y-axis shows −log10 p-values for individual SNPs and the light blue line on the right Y-axis indicates the recombination rates. Pairwise LD (r^2) with the sentinel variant is based on 1000 Genomes European reference samples and is described using the color coding.

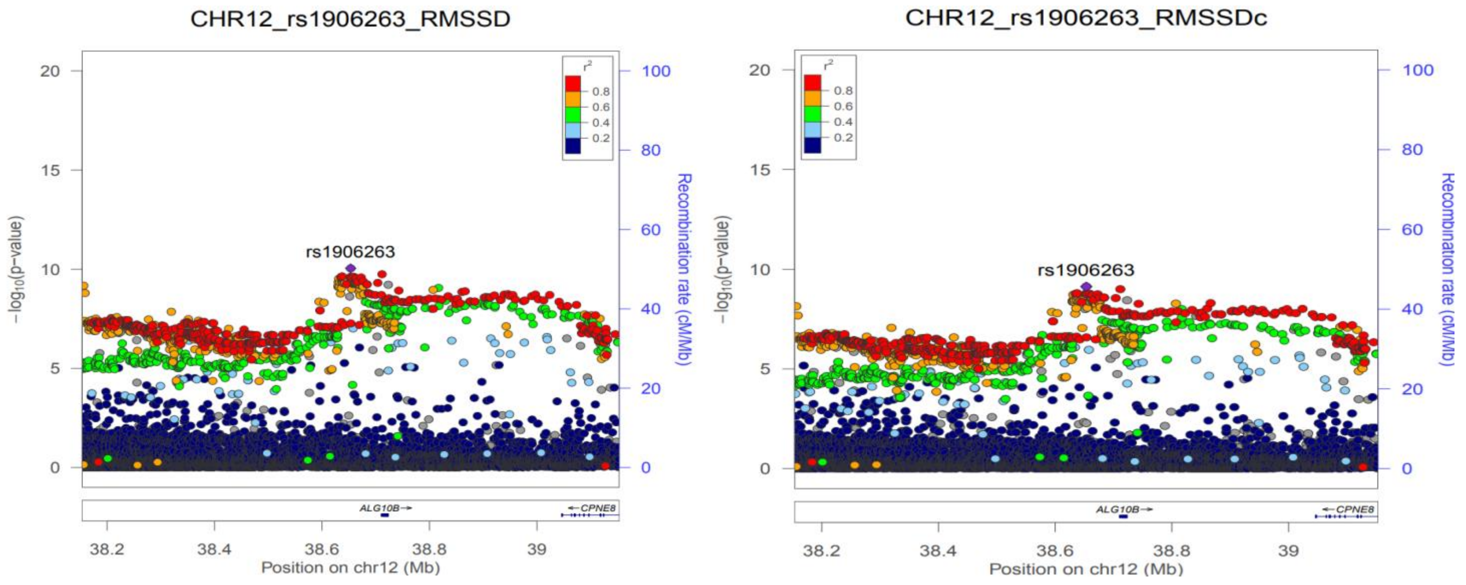

Supplementary Figure 15. Regional association plots for SNP rs1906263. Chromosome number and HRV trait are indicated in the top line. The x-axis depicts a 1Mb region, 500kb either side of the sentinel variant (purple diamond) and the bottom panel show genes located within the region. The left y-axis shows −log10 p-values for individual SNPs and the light blue line on the right Y-axis indicates the recombination rates. Pairwise LD (r^2) with the sentinel variant is based on 1000 Genomes European reference samples and is described using the color coding.

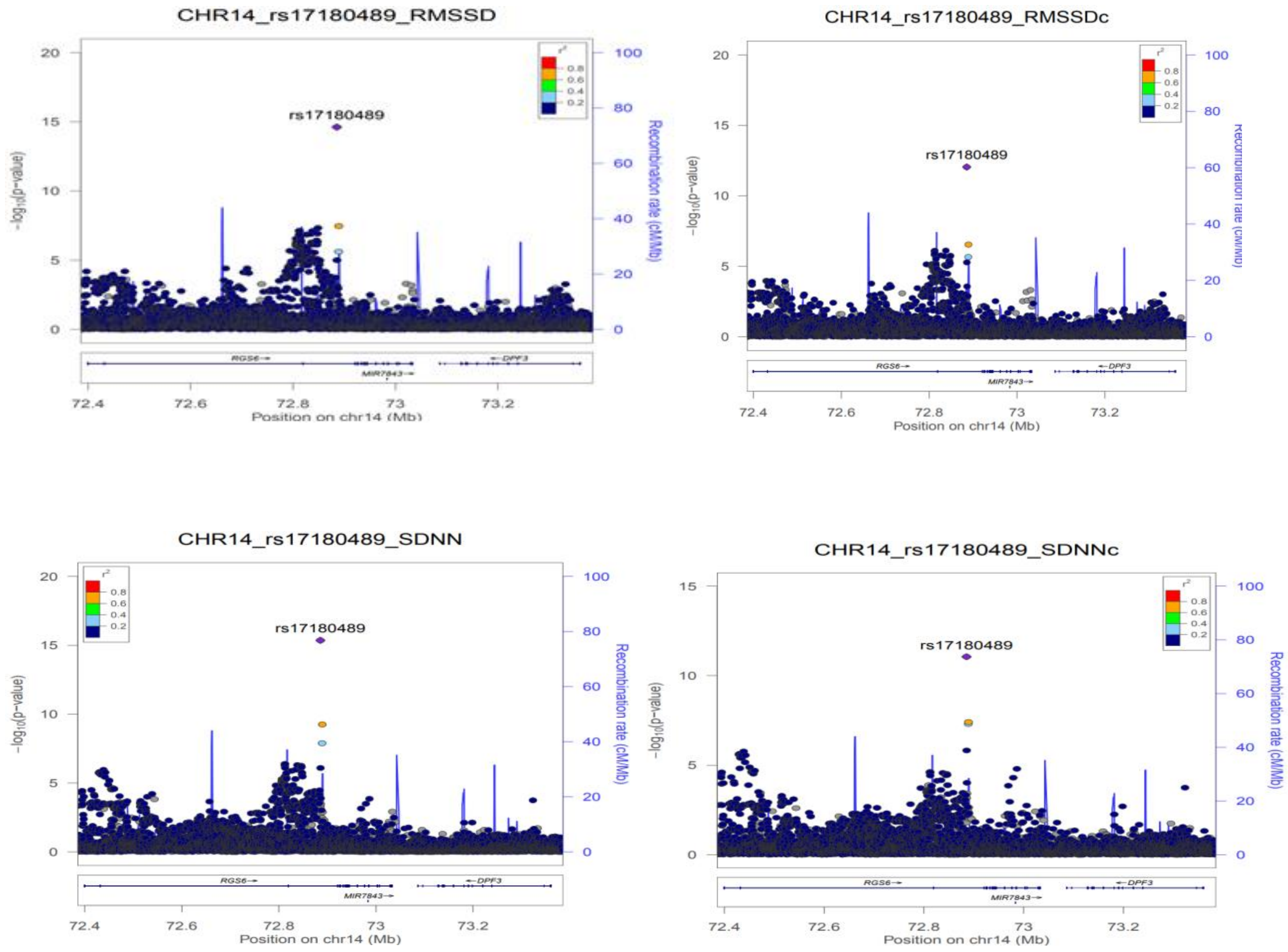

Supplementary Figure 16. Regional association plots for SNP rs17180489. Chromosome number and HRV trait are indicated in the top line. The x-axis depicts a 1Mb region, 500kb either side of the sentinel variant (purple diamond) and the bottom panel show genes located within the region. The left y-axis shows −log10 p-values for individual SNPs and the light blue line on the right Y-axis indicates the recombination rates. Pairwise LD (r^2) with the sentinel variant is based on 1000 Genomes European reference samples and is described using the color coding.

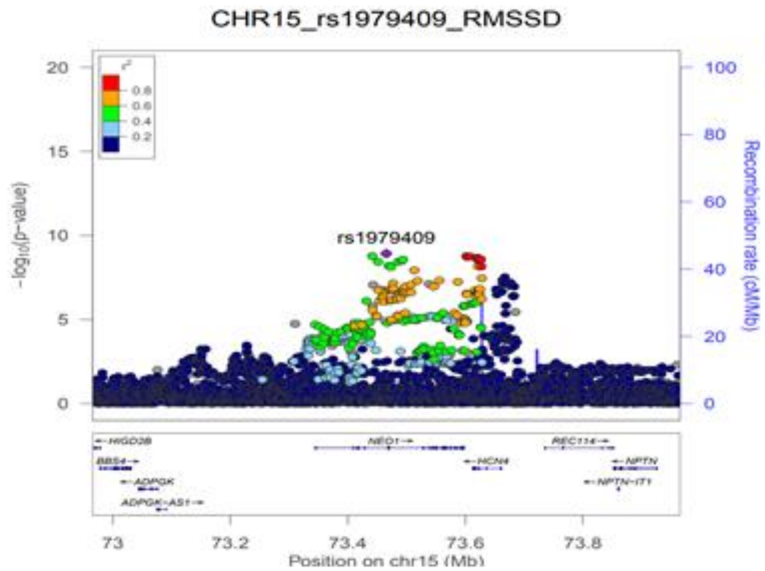

Supplementary Figure 17. Regional association plots for SNP rs1979409. Chromosome number and HRV trait are indicated in the top line. The x-axis depicts a 1Mb region, 500kb either side of the sentinel variant (purple diamond) and the bottom panel show genes located within the region. The left y-axis shows −log10 p-values for individual SNPs and the light blue line on the right Y-axis indicates the recombination rates. Pairwise LD (r^2) with the sentinel variant is based on 1000 Genomes European reference samples and is described using the color coding.

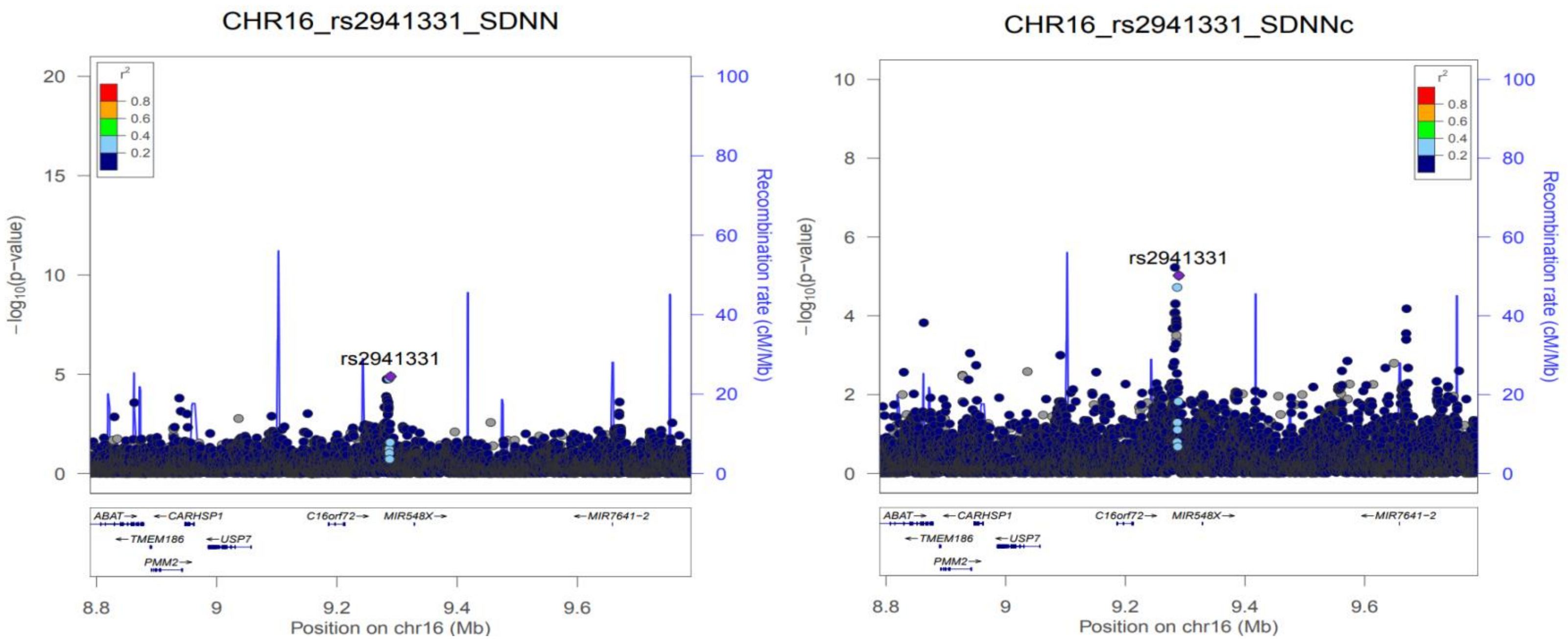

Supplementary Figure 18. Regional association plots for SNP 16:9290009_TA_T. Chromosome number and HRV trait are indicated in the top line. The x-axis depicts a 1Mb region, 500kb either side of the sentinel variant (purple diamond) and the bottom panel show genes located within the region. The left y-axis shows −log10 p-values for individual SNPs and the light blue line on the right Y-axis indicates the recombination rates. Pairwise LD (r^2) with the sentinel variant is based on 1000 Genomes European reference samples and is described using the color coding. Since SNP 16:9290009_TA_T is not available in the 1000 Genomes, we used a proxy SNP, rs2941331, for the regional association plot.

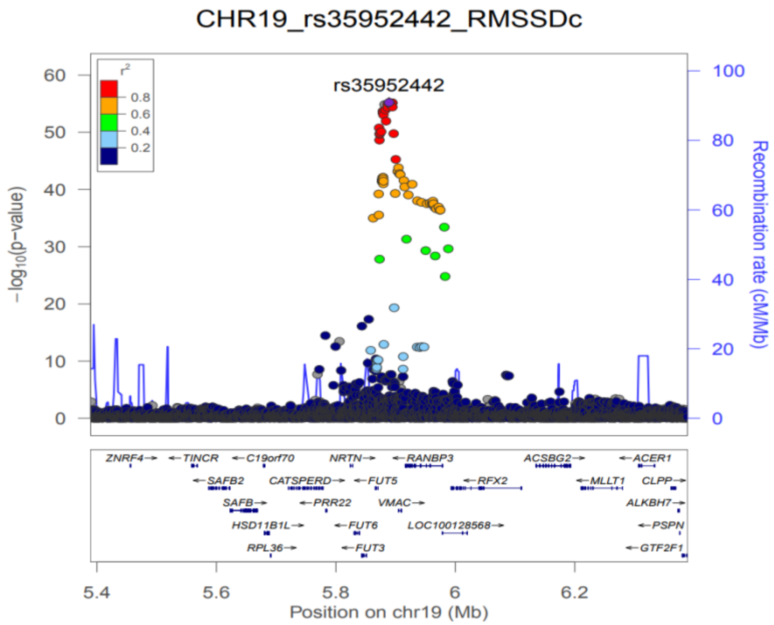

Supplementary Figure 19. Regional association plots for SNP rs35952442. Chromosome number and HRV trait are indicated in the top line. The x-axis depicts a 1Mb region, 500kb either side of the sentinel variant (purple diamond) and the bottom panel show genes located within the region. The left y-axis shows −log10 p-values for individual SNPs and the light blue line on the right Y-axis indicates the recombination rates. Pairwise LD (r^2) with the sentinel variant is based on 1000 Genomes European reference samples and is described using the color coding.

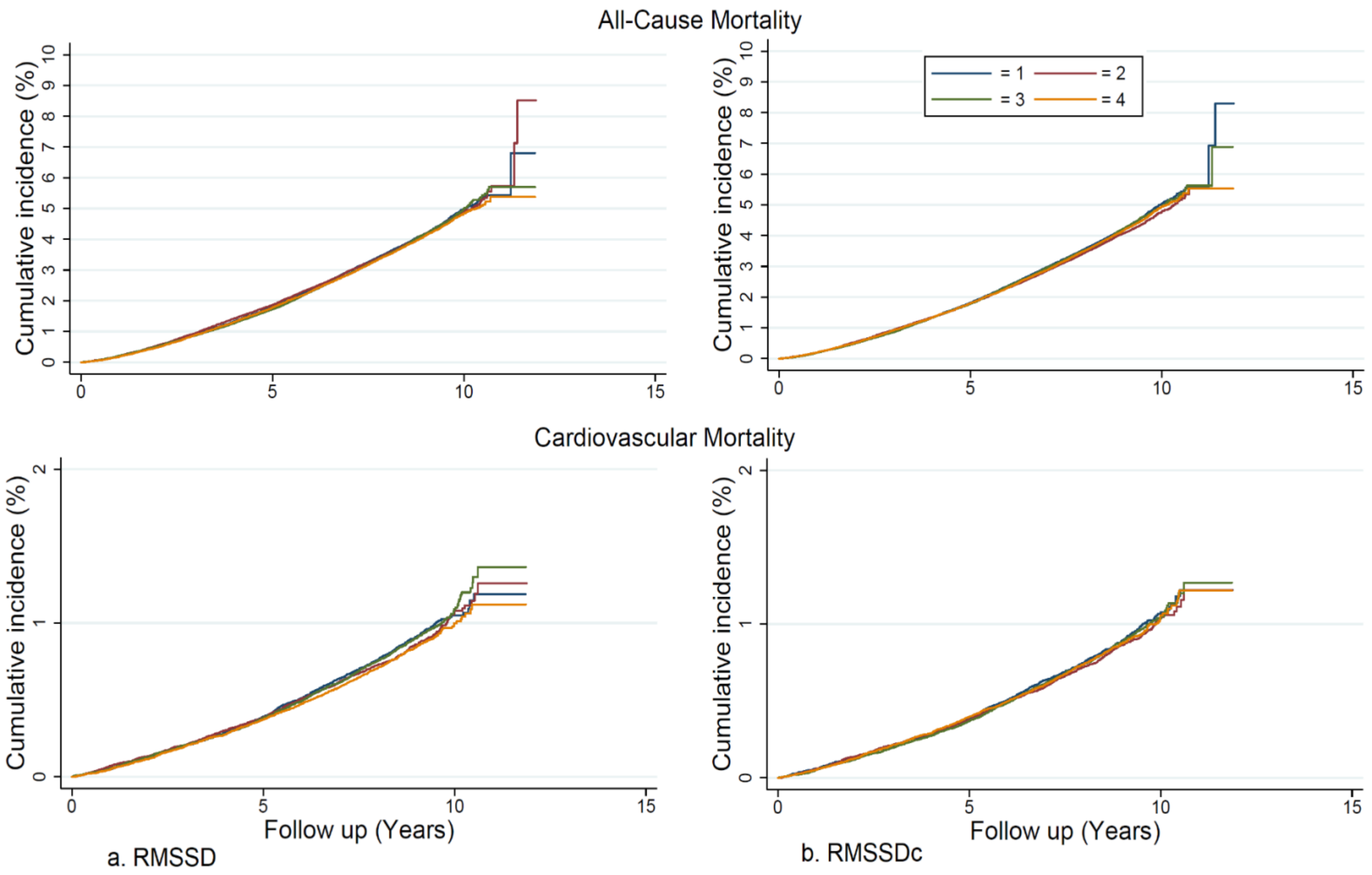

Supplementary Figure 20: Kaplan-Meier curves for risk of all-cause mortality and cardiovascular mortality among participants in quartiles of HRV genetic risk score. a) RMSSD; b) RMSSDc. RMSSD, root mean square of successive differences; RMSSDc, heart rate corrected root mean square of successive differences

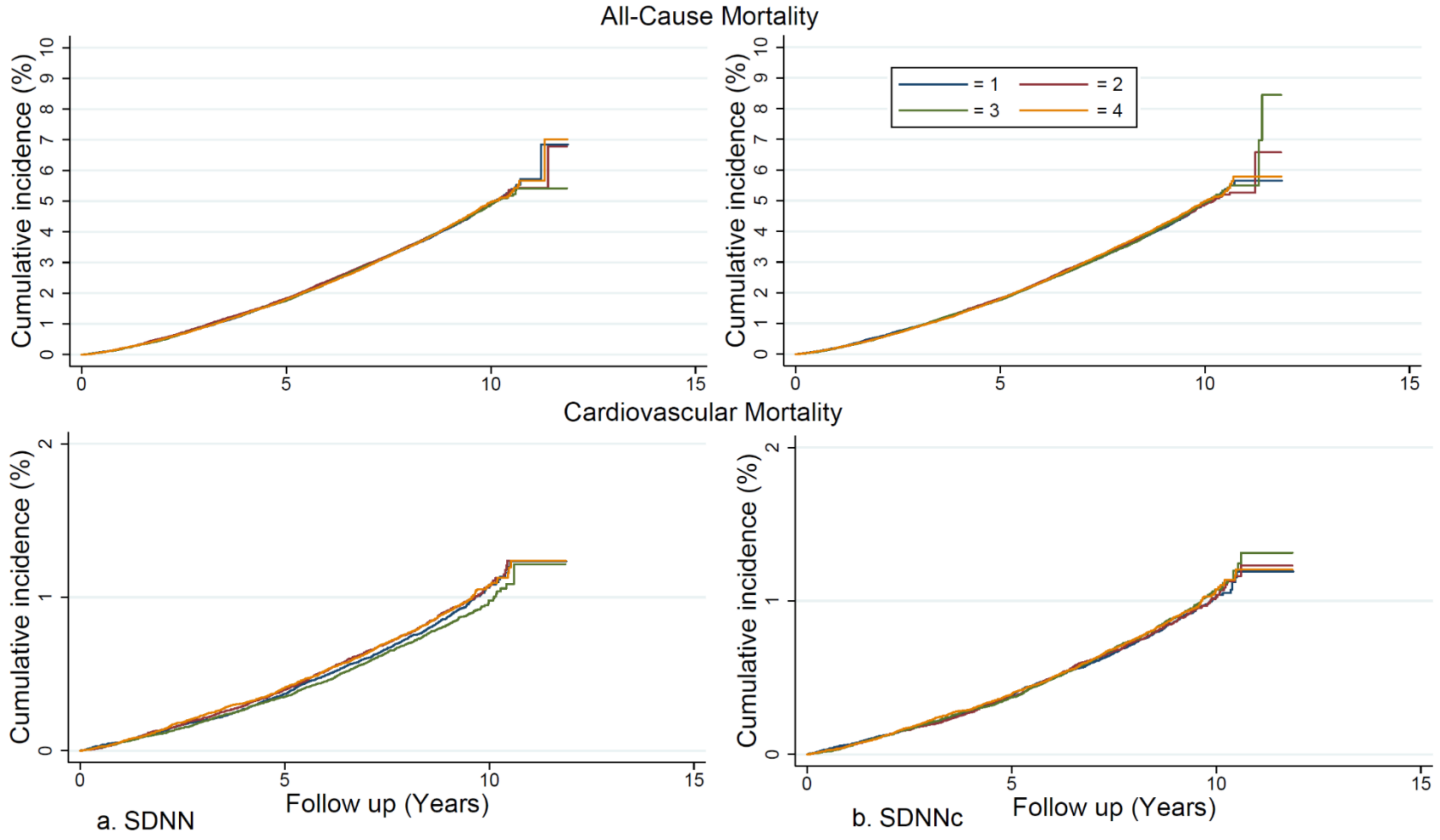

Supplementary Figure 21: Kaplan-Meier curves for risk of all-cause mortality and cardiovascular mortality among participants in quartiles of HRV genetic risk score. a) SDNN; b) SDNNc. SDNN, SD of normal-to-normal intervals; SDNNc, heart rate corrected SD of normal-to-normal intervals

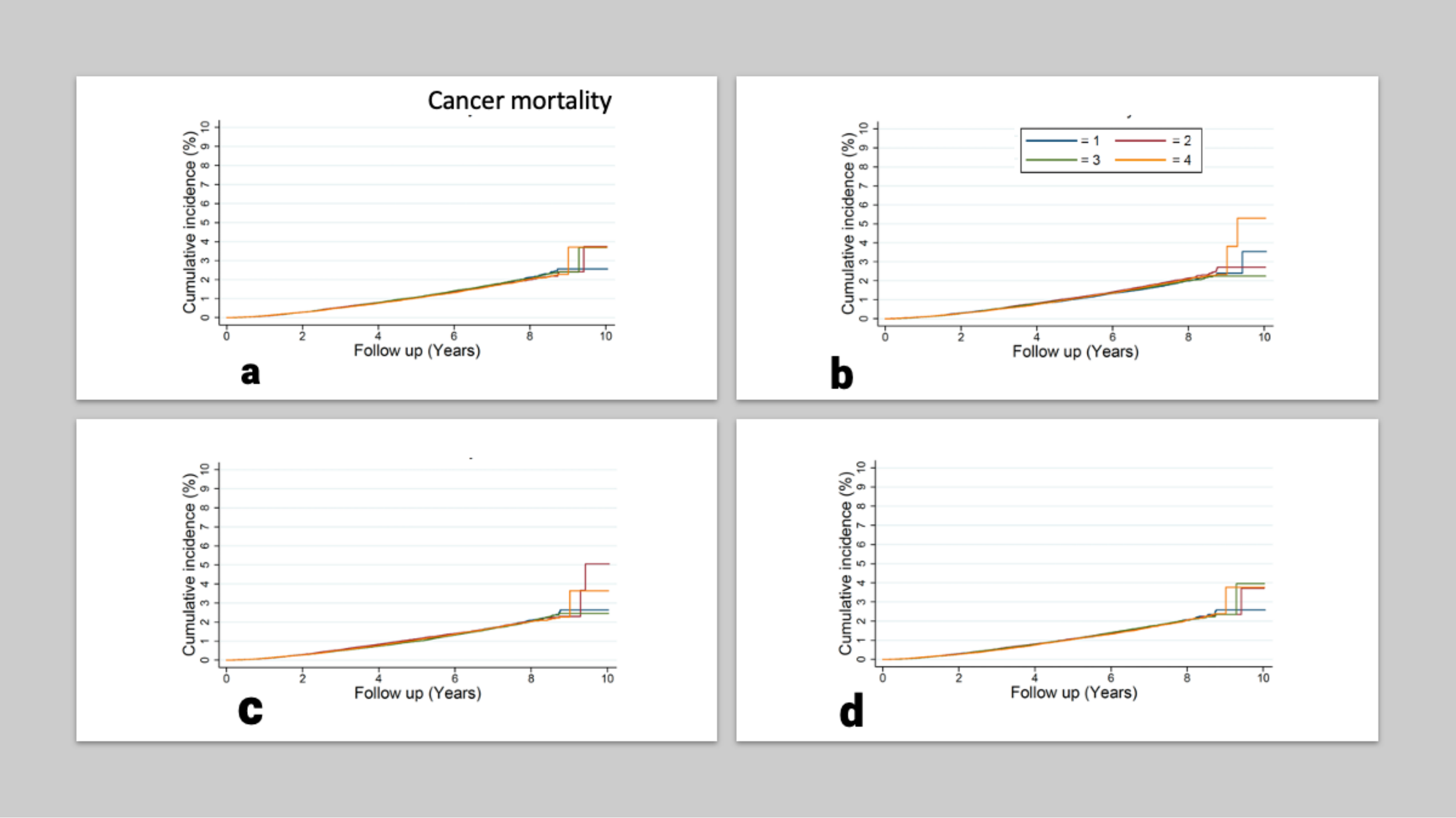

Supplementary Figure 22: Kaplan-Meier curves for risk of cancer mortality among participants in quartiles of HRV genetic risk score. a) RMSSD; b) RMSSDc; c) SDNN; and d) SDNNc. RMSSD, root mean square of successive differences; RMSSDc, heart rate corrected root mean square of successive differences, SDNN, SD of normal-to-normal intervals; SDNNc, heart rate corrected SD of normal-to-normal intervals

Supplementary Note 1: Description of candidate genes at HRV risk loci

***RNF220* (rs156653) Ring finger protein 220:** The sentinel variant at this locus, rs156653, is located in the intronic region of *RNF220* which functions as an E3 ubiquitin ligase, which determines protein target specificity during posttranslational ubiquitination. This gene has also been associated with resting heart rate[1] and heart rate response to post exercise recovery[2,3].

***GNB4* (rs7612445):** Guanine Nucleotide-Binding Protein Subunit Beta-4 on chromosome 3 is a protein coding gene. Heterotrimeric guanine nucleotide-binding proteins (G proteins) are involved as a modulator or transducer in various transmembrane signaling systems, including vagal-muscarinergic signaling underlying HRV, are composed of an alpha, a beta, and a gamma subunit. *GNB4* gene encodes a beta subunit. Beta subunits are important regulators of alpha subunits, as well as of certain signal transduction receptors and effectors like the G protein-gated inwardly rectifying potassium (GIRK) channel. This gene has been also previously reported for atrial fibrillation[4] and diastolic blood pressure[5]. The lead SNP at this locus has been previously associated with heart rate[1,6] and atrial fibrillation[7].

***LINCR-0002* (rs1083698):** is uncharacterized long intergenic non-protein coding RNA gene at chromosome 3 of unknown biological function.

HNRNPA0/ KLHL3* (rs56210945, rs2905583):** Heterogeneous Nuclear Ribonucleoprotein A0 is a protein coding gene located at chromosome 5. Among its related pathways are chromatin regulation / acetylation and gene expression. Gene Ontology annotations related to this gene include nucleic acid binding and RNA binding. The second locus at chromosome 5, *KLHL3,** kelch like family member 3; encodes a full-length protein which has an N-terminal BTB domain followed by a BACK domain and six kelch-like repeats in the C-

terminus. These kelch-like repeats promote substrate ubiquitination of bound proteins via interaction of the BTB domain with the CUL3 (cullin 3) component of a cullin-RING E3 ubiquitin ligase (CRL) complex. The *KLHL3* protein identifies the target of the E3 ubiquitin ligase complex and attaches the complex to it. Complexes containing the *KLHL3* protein tag proteins called WNK1 and WNK4 with ubiquitin. The WNK1 and WNK4 proteins are involved in controlling blood pressure in the body. By regulating the amount of these proteins available, *KLHL3* plays a role in blood pressure control. Mutations in this gene cause pseudo-hypoaldosteronism type II; a rare Mendelian syndrome featuring hypertension, hyperkalemia and metabolic acidosis. An above normal level of potassium can interfere with proper electric signals in cardiac muscle layer and may lead to different types of heart arrhythmias that might potentially lead to a low heart rate variability. The lead SNP at this locus is an intron variant which has not been previously reported. *KLHL3* has been recently reported for heart failure[8].

***PPIL1* (rs236349)**: peptidylprolyl isomerase-like 1 is a previously known gene to be associated with HRV[9] at chromosome 6. Nonetheless, the biological hypothesis to explain the association with HRV is not known. The *PPIL1* gene encodes a component of the major spliceosome complex, which mediates pre-mRNA splicing essential for gene expression and regulation.

***CHRM2* (rs71784944):** cholinergic receptor muscarinic 2 is a protein coding gene at chromosome 7. The muscarinic cholinergic receptors belong to a larger family of G protein-coupled receptors. The functional diversity of these receptors is defined by the binding of acetylcholine to these receptors and includes cellular responses such as adenylate cyclase inhibition, phosphoinositide degeneration, and potassium channel mediation. Muscarinic receptors influence many effects of acetylcholine in the central and peripheral nervous

system. The muscarinic cholinergic receptor 2, located in both pre- and postsynaptically, and act as auto-receptors, a category of receptor that restricts ongoing synaptic activity by constraining the release or synthesis of a set neurotransmitter, is involved in mediation of bradycardia and a decrease in cardiac contractility. Among the different tissues, *CHRM2* is found in the sinoatrial node of the heart, and thus *CHRM2* affects heart rate and cardiac function. This gene has been previously reported to be associated with heart rate[1,6], heart rate response to recovery post exercise[2,3]and pulse pressure measurement[5,10].

***GNG11* (rs756675674, rs180251, rs180244):** Guanine nucleotide-binding protein, gamma-11, which is also a previously known HRV gene[9], is located at chromosome 7. *GNG11* encodes the γ11 subunit of the same heterotrimeric G protein complex that *GNB4* is part of, which is involved as a modulator or transducer in various transmembrane signaling systems including vagal-muscarinergic signaling underlying HRV. This gene has been also reported in association with heart rate and heart response to exercise[1,2].

***KCNJ5* (rs76097649, rs7102584):** potassium inwardly rectifying channel subfamily J member 5, this gene encodes an integral membrane protein which belongs to one of seven subfamilies of inward-rectifier potassium channel proteins called potassium channel subfamily J. The encoded protein is a subunit of the potassium channel which is homotetrameric. It is controlled by G-proteins and has a greater tendency to allow potassium to flow into a cell rather than out of a cell. It enables the transmembrane transfer of a potassium ion by a voltage-gated channel through the plasma membrane of an atrial cardiomyocyte contributing to the repolarization phase of an action potential. Naturally occurring mutations in this gene are associated with Long QT syndrome[11] and familial hyperaldostronism[12]. *KCNJ5* is a previously reported gene for resting heart rate[1] atrial fibrillation[4,7,13] and diastolic blood pressure[10]. One of lead SNPs at this

locus, rs76097649, has been previous reported for atrial fibrillation[4,7]. The second lead SNP at this locus, rs7102584, is a non-synonymous SNP.

***MED13L* (rs802122):** mediator of RNA polymerase II transcription subunit 13-like is a component of the mediator complex, a coactivator involved in the regulated transcription of nearly all RNA polymerase II-dependent genes. Mediator functions as a bridge to convey information from gene-specific regulatory proteins to the basal RNA polymerase II transcription machinery.

***LINC00477* (rs4963772):** long intergenic non-protein coding RNA 477, located at chromosome 12, has no known biological function however the locus has also been associated with HRV[9].

***SYT10* (rs6488162):** synaptotagmin-10, a locus found at chromosome 12, encodes a Ca^{2+} sensor that has a role in the regulation of calcium dependent exocytosis, including calcium regulated release of neurotransmitter from presynaptic nerve terminals[14]. SYT10 might play an important role in the regulation of HRV, as it was found to be associated with heart rate[1], heart rate increase[2], and HRV[9] as well.

***ALG10B* (rs1906263, rs35861884**): Alpha-1,2-Glucosyltransferase B) is a Protein Coding gene. It is a plasma membrane-associated protein that appears to function as a regulatory subunit of the cardiac potassium channel. *ALG10B* has been previously reported to be associated with chronotype/sleep duration[15] circadian rhythm[16].

***RGS6* (rs17180489):** regulator of G-protein signaling 6, located at chromosome 14, is a well know gene for heart rate and cardiac rhythm[1,2,9]. *RGS6* plays a role in the parasympathetic regulation of the heart and decreases muscarinic type 2 receptor signaling in the sinoatrial node by rapidly terminating Gβγ signalling[17].

***NEO1* (rs1979409):** Neogenin 1 is a protein coding gene at chromosome 15 that encodes a cell surface protein that is a member of the immunoglobulin superfamily. It is a multi-functional cell surface receptor regulating cell adhesion in many diverse developmental processes, including neural tube and mammary gland formation, myogenesis and angiogenesis. Although the biologic mechanism is not known, NEO1 has been previously reported to be associated with HRV[9].

***C16orf72* (16:9290009_TA_T):** Chromosome 16 Open Reading Frame 72 is a protein coding gene of unknown biological function.

***NDUFA11* (rs201334918, rs35952442, rs12974991)**: NADH: ubiquinone oxidoreductase subunit A11, at chromosome 19, encodes a subunit of the membrane-bound mitochondrial complex I. A recent study on mice[18] has shown that downregulation of NDUFA11 reduced ATP production and increased mitochondria reactive oxygen species production in cardiac mitochondria. This gene has been also previously reported for HRV[9].

Supplementary References

1. Eppinga RN, Hagemeijer Y, Burgess S, et al. Identification of genomic loci associated with resting heart rate and shared genetic

predictors with all-cause mortality. *Nat Genet*. 2016. doi:10.1038/ng.3708

2. Verweij N, Van De Vegte YJ, Van Der Harst P. Genetic study links components of the autonomous nervous system to heart-rate profile during exercise. *Nat Commun*. 2018. doi:10.1038/s41467-018-03395-6

3. Ramírez J, Duijvenboden S Van, Ntalla I, et al. Thirty loci identified for heart rate response to exercise and recovery implicate autonomic nervous system. *Nat Commun*. 2018. doi:10.1038/s41467-018-04148-1

4. Roselli C, Chaffin MD, Weng LC, et al. Multi-ethnic genome-wide association study for atrial fibrillation. *Nat Genet*. 2018. doi:10.1038/s41588-018-0133-9

5. Evangelou E, Warren HR, Mosen-Ansorena D, et al. Genetic analysis of over 1 million people identifies 535 new loci associated with blood pressure traits. *Nat Genet*. 2018. doi:10.1038/s41588-018-0205-x

6. Den Hoed M, Eijgelsheim M, Esko T, et al. Identification of heart rate-associated loci and their effects on cardiac conduction and rhythm disorders. *Nat Genet*. 2013. doi:10.1038/ng.2610

7. Nielsen JB, Thorolfsdottir RB, Fritsche LG, et al. Biobank-driven genomic discovery yields new insight into atrial fibrillation biology. *Nat Genet*. 2018. doi:10.1038/s41588-018-0171-3

8. Shah S, Henry A, Roselli C, et al. Genome-wide association and Mendelian randomisation analysis provide insights into the pathogenesis of heart failure. *Nat Commun*. 2020. doi:10.1038/s41467-019-13690-5

9. Nolte IM, Munoz ML, Tragante V, et al. Genetic loci associated with heart rate variability and their effects on cardiac disease risk. *Nat Commun*. 2017;8. doi:10.1038/ncomms15805

10. Warren HR, Evangelou E, Cabrera CP, et al. Genome-wide association analysis identifies novel blood pressure loci and offers biological insights into cardiovascular risk. *Nat Genet*. 2017. doi:10.1038/ng.3768

11. Yang Y, Yang Y, Liang B, et al. Identification of a Kir3.4 Mutation in Congenital Long QT Syndrome. *Am J Hum Genet*. 2010. doi:10.1016/j.ajhg.2010.04.017

12. Charmandari E, Sertedaki A, Kino T, et al. A novel point mutation in the KCNJ5 gene causing primary hyperaldosteronism and early-onset autosomal dominant hypertension. *J Clin Endocrinol Metab*. 2012. doi:10.1210/jc.2012-1334

13. Christophersen IE, Rienstra M, Roselli C, et al. Large-scale analyses of common and rare variants identify 12 new loci associated with atrial fibrillation. *Nat Genet*. 2017. doi:10.1038/ng.3843

14. Cao P, Maximov A, Südhof TC. Activity-dependent IGF-1 exocytosis is controlled by the Ca 2+-sensor synaptotagmin-10. *Cell*. 2011. doi:10.1016/j.cell.2011.03.034

15. Dashti HS, Jones SE, Wood AR, et al. Genome-wide association study identifies genetic loci for self-reported habitual sleep duration supported by accelerometer-derived estimates. *Nat Commun*. 2019. doi:10.1038/s41467-019-08917-4

16. Jones SE, Lane JM, Wood AR, et al. Genome-wide association analyses of chronotype in 697,828 individuals provides insights

into circadian rhythms. *Nat Commun*. 2019. doi:10.1038/s41467-018-08259-7

17. Yang J, Huang J, Maity B, et al. RGS6, a modulator of parasympathetic activation in heart. *Circ Res*. 2010. doi:10.1161/CIRCRESAHA.110.224220

18. Jang S, Javadov S. Elucidating the contribution of ETC complexes I and II to the respirasome formation in cardiac mitochondria. *Sci Rep*. 2018. doi:10.1038/s41598-018-36040-9
