# Supplementary material for: Phenotypic but not genetically predicted heart rate variability associated with all-cause mortality
Source: Commun Biol. 2023 Oct 6;6:1013. doi: 10.1038/s42003-023-05376-y (PMC10558565; doi:10.1038/s42003-023-05376-y)
Supplement: Supplementary file 3 — Description of Additional Supplementary Files [file 42003_2023_5376_MOESM3_ESM.pdf]

Description of Additional Supplementary Files

File name: Supplementary Data 1
Description: Sex stratified characteristics of the UK Biobank study participants.

File name: Supplementary Data 2
Description: Phenotypic associations of HRV traits with all-cause mortality and cardiovascular mortality.

File name: Supplementary Data 3
Description: Associations of heart rate variability traits with all-cause mortality and cardiovascular mortality.

File name: Supplementary Data 4
Description: Associations of genetically predicted heart rate variability with all-cause mortality and cardiovascular mortality.

File name: Supplementary Data 5
Description: Associations of the quartiles of the genetic risk score for heart rate variability with all-cause mortality, cardiovascular mortality and cancer mortality.

File name: Supplementary Data 6
Description: In-silico sequencing and pleiotropy analysis of significant genetic variants for heart rate variability.

File name: Supplementary Data 7
Description: Previously reported loci for heart rate variability in a meta-analysis of individuals of European ancestry.

File name: Supplementary Data 8
Description: SNP based heritability and genetic correlation of HRV traits.

File name: Supplementary Data 9
Description: Genetic correlation by cross-trait LD score regression.

File name: Supplementary Data 10
Description: Power calculations using mRnd.

File name: Supplementary Data 11
Description: Associations of heart rate variability traits with cancer mortality.

File name: Supplementary Data 12
Description: Associations of heart rate variability traits with all-cause mortality and cardiovascular mortality after exclusion of participants who died within 1 and 2 years after baseline.

File name: Supplementary Data 13
Description: Comparison of the effect size and p-values of genome-wide significant variants after exclusion of participants who died within 1 and 2 years after baseline.

File name: Supplementary Data 14
Description: Look-ups of genome-wide significant variants in a previous meta-analysis of individuals of European ancestry.
