# Supplementary material for: Phenotypic but not genetically predicted heart rate variability associated with all-cause mortality
Source: Commun Biol. 2023 Oct 6;6:1013. doi: 10.1038/s42003-023-05376-y (PMC10558565; doi:10.1038/s42003-023-05376-y)
Supplement: Supplementary file 5 — Reporting summary [file 42003_2023_5376_MOESM5_ESM.pdf]

nature portfolio

Corresponding author(s): Harold Snieder

Last updated by author(s): Sep 18, 2023

Reporting Summary

Nature Portfolio wishes to improve the reproducibility of the work that we publish. This form provides structure for consistency and transparency in reporting. For further information on Nature Portfolio policies, see our Editorial Policies and the Editorial Policy Checklist.

Statistics

For all statistical analyses, confirm that the following items are present in the figure legend, table legend, main text, or Methods section.

n/a	Confirmed	
☐	☒	The exact sample size (*n*) for each experimental group/condition, given as a discrete number and unit of measurement
☐	☒	A statement on whether measurements were taken from distinct samples or whether the same sample was measured repeatedly
☐	☒	The statistical test(s) used AND whether they are one- or two-sided<br>*Only common tests should be described solely by name; describe more complex techniques in the Methods section.*
☐	☒	A description of all covariates tested
☐	☒	A description of any assumptions or corrections, such as tests of normality and adjustment for multiple comparisons
☐	☒	A full description of the statistical parameters including central tendency (e.g. means) or other basic estimates (e.g. regression coefficient) AND variation (e.g. standard deviation) or associated estimates of uncertainty (e.g. confidence intervals)
☐	☒	For null hypothesis testing, the test statistic (e.g. *F*, *t*, *r*) with confidence intervals, effect sizes, degrees of freedom and *P* value noted<br>*Give P values as exact values whenever suitable.*
☒	☐	For Bayesian analysis, information on the choice of priors and Markov chain Monte Carlo settings
☒	☐	For hierarchical and complex designs, identification of the appropriate level for tests and full reporting of outcomes
☐	☒	Estimates of effect sizes (e.g. Cohen's *d*, Pearson's *r*), indicating how they were calculated

Our web collection on statistics for biologists contains articles on many of the points above.

Software and code

Policy information about availability of computer code

Data collection	AM-USB 6.5, Cardiosoft v6.51 was used for ECG recording
Data analysis	BOLT-LMM, GWASinpecter R package, PLINK 1.9, LD Score Regression , STATA 14, ANNOVAR software, Sorting Intolerant From Tolerant (SIFT) and Polymorphism Phenotyping (PolyPhen)

For manuscripts utilizing custom algorithms or software that are central to the research but not yet described in published literature, software must be made available to editors and reviewers. We strongly encourage code deposition in a community repository (e.g. GitHub). See the Nature Portfolio guidelines for submitting code & software for further information.

Data

Policy information about availability of data

All manuscripts must include a data availability statement. This statement should provide the following information, where applicable:

- Accession codes, unique identifiers, or web links for publicly available datasets
- A description of any restrictions on data availability
- For clinical datasets or third party data, please ensure that the statement adheres to our policy

Access of raw data is restricted to protect the privacy of participants, but it can be requested via UK Biobank (www.ukbiobank.ac.uk). Summary statistics will be uploaded to the GWAS catalog upon acceptance to make them publicly available.

Human research participants

Policy information about studies involving human research participants and Sex and Gender in Research.

Reporting on sex and gender	In our analyses we used sex (reported by participants) as a covariate to adjust our analyses where more than half of the study participants were females.
Population characteristics	age, history of prevalent diseases and medication use like anti-depressants were also part of our covariates which we considered in this manuscript
Recruitment	This study uses data from the UK Biobank, a prospective cohort study of 503,325 individuals from the United Kingdom with an age range of 40-69 that were recruited between 2006 and 2010. Participant recruitment was based on invitations being mailed to people be whose contact details were obtained from National Health Service (NHS) central registers. Participants spent an average of about two and a half hours at the recruitment visit. All gave broad consent to use of their anonymised data and samples for any health-related research, to be re-contacted for further substudies, and for UK Biobank to access their health-related records. Given that the sample size is huge and the recruitment was across the country, the risk of selection bias is considered small
Ethics oversight	The Northwest Multi-Centre Research Ethics Committee approved the UK Biobank study

Note that full information on the approval of the study protocol must also be provided in the manuscript.

Field-specific reporting

Please select the one below that is the best fit for your research. If you are not sure, read the appropriate sections before making your selection.

☒ Life sciences ☐ Behavioural & social sciences ☐ Ecological, evolutionary & environmental sciences

For a reference copy of the document with all sections, see nature.com/documents/nr-reporting-summary-flat.pdf

Life sciences study design

All studies must disclose on these points even when the disclosure is negative.

Sample size	The sample size was pre-determined with the number of participants who had genotype data and electrocardiogram recordings. The sample size was more than 46,000, which was adequately to test our associations
Data exclusions	Non-Europeans (n=5,352) were excluded to get a homogeneous population. Participants were also excluded if they had missing or poor quality genotype data, missing covariates. We also excluded participants with a diagnosis of angina, myocardial infarction, heart failure, or used anti-depressant medication, digoxin, atropine, or acetylcholinesterase since these are known to influence our outcome measures
Replication	For the genetic analyses, we did a lookup of the identified genome-wide significant genetic variants in meta-analyzed GWAS data of 20 cohorts of European ancestry in up to 28,700 participants. An association was considered replicated if the direction of effect was consistent and the one-sided replication p-value was < 0.05 where almost all the association signals had a pvalue less than 0.05. We also evaluated replication for a more conservative Bonferroni corrected one-sided threshold (p<0.00294) corrected for the total number of 17 independent SNPs (0.05/17)
Randomization	Randomization was not applicable as our test was based on the association of genetic variants (the random assignment of genetic variants from parents to offspring is fundamental (Mendel's law) and random) and Heart rate variability.
Blinding	blinding was not applicable for our study

Reporting for specific materials, systems and methods

We require information from authors about some types of materials, experimental systems and methods used in many studies. Here, indicate whether each material, system or method listed is relevant to your study. If you are not sure if a list item applies to your research, read the appropriate section before selecting a response.

Materials & experimental systems

n/a	Involved in the study
☒	☐ Antibodies
☒	☐ Eukaryotic cell lines
☒	☐ Palaeontology and archaeology
☒	☐ Animals and other organisms
☒	☐ Clinical data
☒	☐ Dual use research of concern

Methods

n/a	Involved in the study
☒	☐ ChIP-seq
☒	☐ Flow cytometry
☒	☐ MRI-based neuroimaging
